# Supplementary material for: Stepwise DNA unwinding gates TnpB genome-editing activity
Source: bioRxiv. 2026 Jan 9:2026.01.09.698545. Preprint. [Version 1] doi: 10.64898/2026.01.09.698545 (PMC12803034; doi:10.64898/2026.01.09.698545)
Supplement: Supplement 1 [file media-1.pdf]

Supplemental Document for

Stepwise DNA unwinding gates TnpB genome-editing activity

Zehan Zhou[1,2†], Iren Saffarian-Deemyad[3†], Honglue Shi[1,4,5†], Trevor Weiss[6†], Muhammad Moez ur-Rehman[1,2], Kamakshi Vohra[1,5,‡], Petr Skopintsev[1,5], Peter H. Yoon[1,2], Marena I. Trinidad[1,7], Conner Langeberg[1,5], Maris Kamalu[6], Jasmine Amerasekera[6], Erin E. Doherty[1,5], Kevin D.P. Aris[8,9,§], Noor Al-Sayyad[3,8], Brittney W. Thornton[1,2], Rachel F. Weissman[1,2], Kevin M. Wasko[1,2], Isabel Esain-Garcia[1,4], Evan C. DeTurk[1,5,#], David F. Savage[1,2,4,5], Steven E. Jacobsen[6,10]*, Zev Bryant[8,11]*, Jennifer A. Doudna[1,2,4,5,12-17]*

[1]Innovative Genomics Institute, University of California, Berkeley, CA, USA, 94720

[2]Department of Molecular and Cell Biology, University of California, Berkeley, CA, USA, 94720

[3]Department of Physics, Stanford University, Stanford, CA, USA, 94305

[4]Howard Hughes Medical Institute, University of California, Berkeley, CA, USA, 94720

[5]California Institute for Quantitative Biosciences (QB3), University of California, Berkeley, Berkeley, CA, USA, 94720

[6]Department of Molecular, Cell and Developmental Biology, University of California at Los Angeles, Los Angeles, CA, USA, 90095

[7]University of California, Berkeley-University of California, San Francisco Graduate Program in Bioengineering, University of California, Berkeley, Berkeley, CA, USA, 94720

[8]Department of Bioengineering, Stanford University, Stanford, CA, USA, 94305

[9]Biophysics Program, Stanford University, Stanford, CA, USA, 94305

[10]Howard Hughes Medical Institute, University of California at Los Angeles, Los Angeles, CA, USA, 90095

[11]Department of Structural Biology, Stanford University Medical Center, Stanford, CA, USA, 94305

[12]Li Ka Shing Center for Genomic Engineering, University of California, Berkeley, Berkeley, CA, USA, 94720

[13]Department of Chemistry, University of California, Berkeley, Berkeley, CA, USA, 94720

[14]Molecular Biophysics and Integrated Bioimaging Division, Lawrence Berkeley National Laboratory, Berkeley, CA, USA, 94720

[15]Gladstone Institute of Data Science and Biotechnology, San Francisco, CA, USA, 94158

[16]Gladstone-UCSF Institute of Genomic Immunology, San Francisco, CA, USA, 94158

[17]Lead contact

[†]These authors contributed equally

[‡]Present Address: The Biochemistry, Quantitative Biology, Biophysics and Structural Biology (BQBS) Track, Yale University, New Haven, CT, USA, 06511

[§]Present Address: Department of Bioengineering, University of California, Berkeley, Berkeley, CA, USA, 94720

[#]Present Address: Department of History and Philosophy of Science, University of Cambridge, Cambridge, UK

*Correspondence: doudna@berkeley.edu, zevry@stanford.edu, jacobsen@ucla.edu

Supplemental Figures

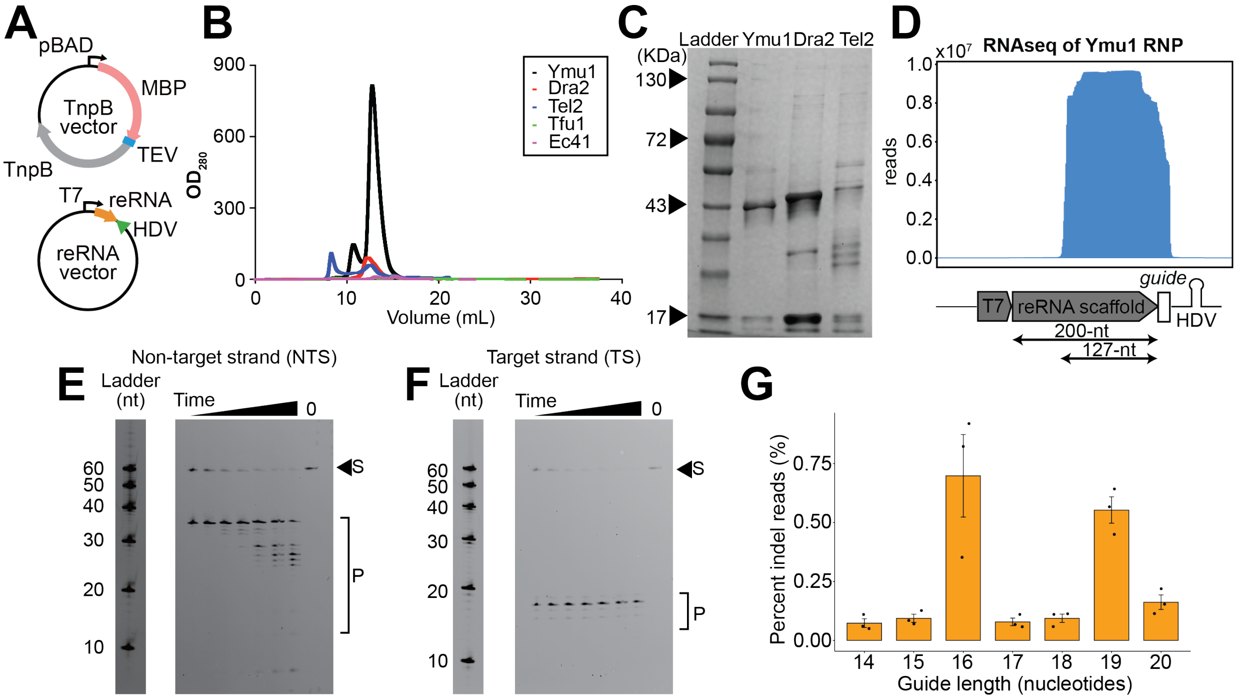

Fig. S1. Additional characterization of Ymu1 TnpB and guide configuration (related to Fig. 1). (A) Schematic of the bacterial expression system used for TnpB ribonucleoprotein (RNP) production. TnpB protein is expressed from an arabinose-inducible promoter, while the reRNA is expressed from a separate plasmid under a T7 promoter and processed by a downstream HDV ribozyme. **(B)** Size-exclusion chromatography traces of five TnpB orthologs (Ymu1, Dra2, Tel2, Tfu1, Ec41). Absorbance at 280 nm (y-axis) is plotted against elution volume (x-axis, mL). **(C)** SDS-PAGE analysis of purified TnpB orthologs corresponding to panel A, confirming protein homogeneity following affinity and size-exclusion purification. **(D)** RNA-seq analysis of Ymu1 reRNA expressed in E. coli from a T7 promoter using a 200-nt reRNA scaffold. **(E-F)** Representative denaturing gels of Target 1 cleavage by reconstituted Ymu1 RNP, showing **(E)** non-target strand (NTS) and **(F)** target strand (TS) cleavage. The uncleaved substrate (S) and cleavage product (P) are indicated. **(G)** Guide-length screen in *Arabidopsis* protoplasts using a single-transcript TnpB-reRNA-HDV design with the WT TnpB and 127-nt reRNA. The percentage of indel reads (y-axis) at the endogenous PDS3 g2 target site using guide length ranging from 14-20 nts (x-axis). Bars represent mean ± SEM (n = 3 biological replicates).

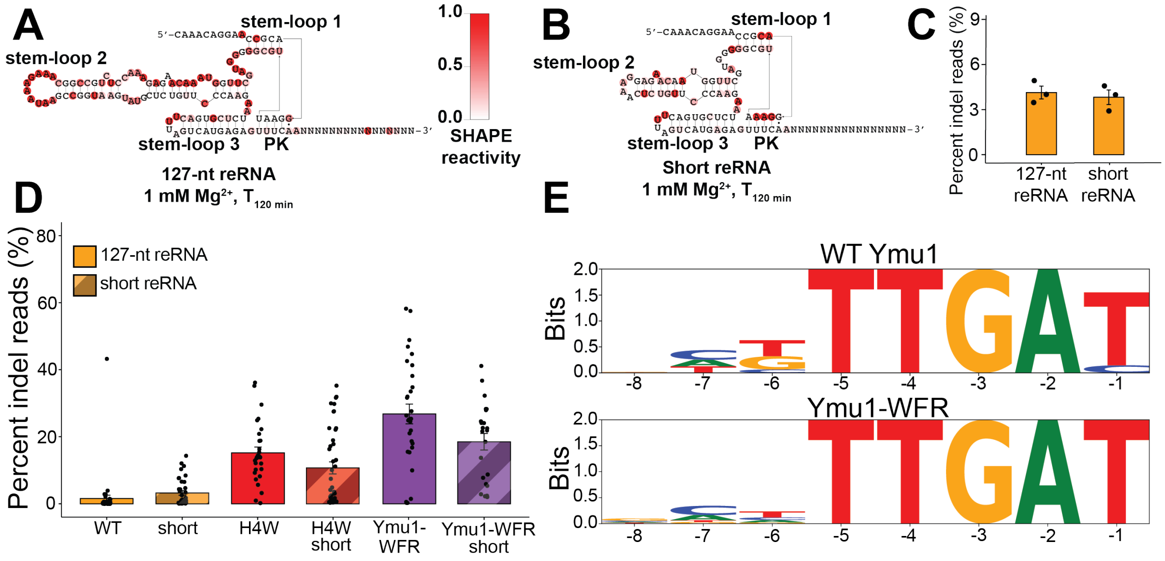

Fig. S2. Structural and functional analysis of Ymu1 reRNA and scaffold variants (related to Fig. 2). **(A-B)** SHAPE-MaP reactivity profile for the **(A)** 127-nt reRNA and **(B)** short reRNA scaffold. Reactivities are mapped onto the predicted secondary structure, with major stem-loop elements and pseudoknot (PK) indicated. **(C)** Protoplast editing efficiencies comparing the 127-nt and short reRNA scaffolds using WT Ymu1. Percentage of indel reads (y-axis) obtained from amplicon sequencing the *Arabidopsis* PDS3 g2 site (x-axis). Bars represent mean ± SEM (n = 3 biological replicates). **(D)** Comparison of T1 whole-plant editing efficiencies targeting *Arabidopsis* PDS3 g2 using full-length versus short reRNA scaffolds. Bar plot shows percentage of indel reads (mean ± SEM). **(E)** Bacterial TAM-discovery assay for WT Ymu1 and Ymu1-WFR. Sequence logos summarize nucleotide enrichment adjacent to the target site.

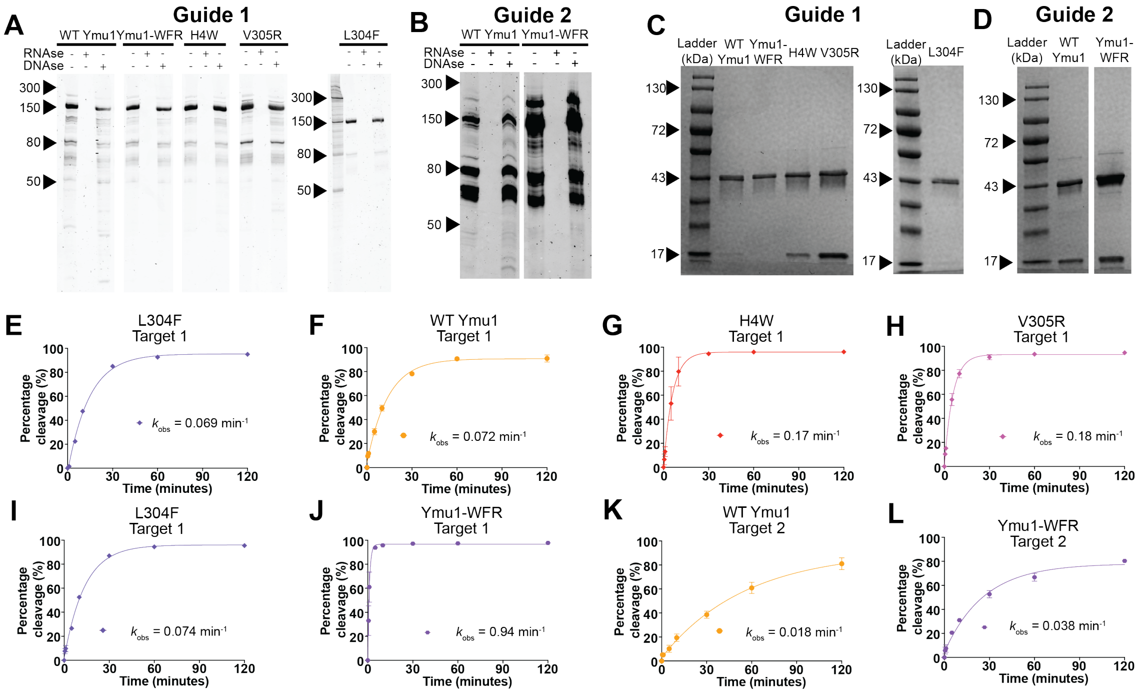

Fig. S3. Additional cleavage assays and biochemical quality controls (related to Fig. 3) (A-B) Denaturing PAGE analysis of the reRNA homogeneity for RNP samples used in cleavage assays targeting **(A)** Target 1 and **(B)** Target 2. Gels are shown under untreated, RNase-treated, and DNase-treated conditions. **(C-D)** SDS-PAGE analysis of the TnpB protein homogeneity RNP samples used in cleavage assays targeting **(C)** Target 1 and **(D)** Target 2. **(E)** NTS cleavage profiles for L304F (Target 1). **(F-L)** TS cleavage profiles for Ymu1 TnpB variants. Percentage of cleaved substrate (y-axis) is plotted over time (x-axis). Each point represents mean ± SD (n = 3 independent reactions); k_{obs} values reflect the mean from three independent mono-exponential fits on each reaction time course. **(F)** WT Ymu1 (Target 1). **(G)** H4W (Target 1). **(H)** V305R (Target 1). **(I)** L304F (Target 1). **(J)** Ymu1-WFR (Target 1). **(K)** WT Ymu1 (Target 2) **(L)** Ymu1-WFR (Target 2)**.**

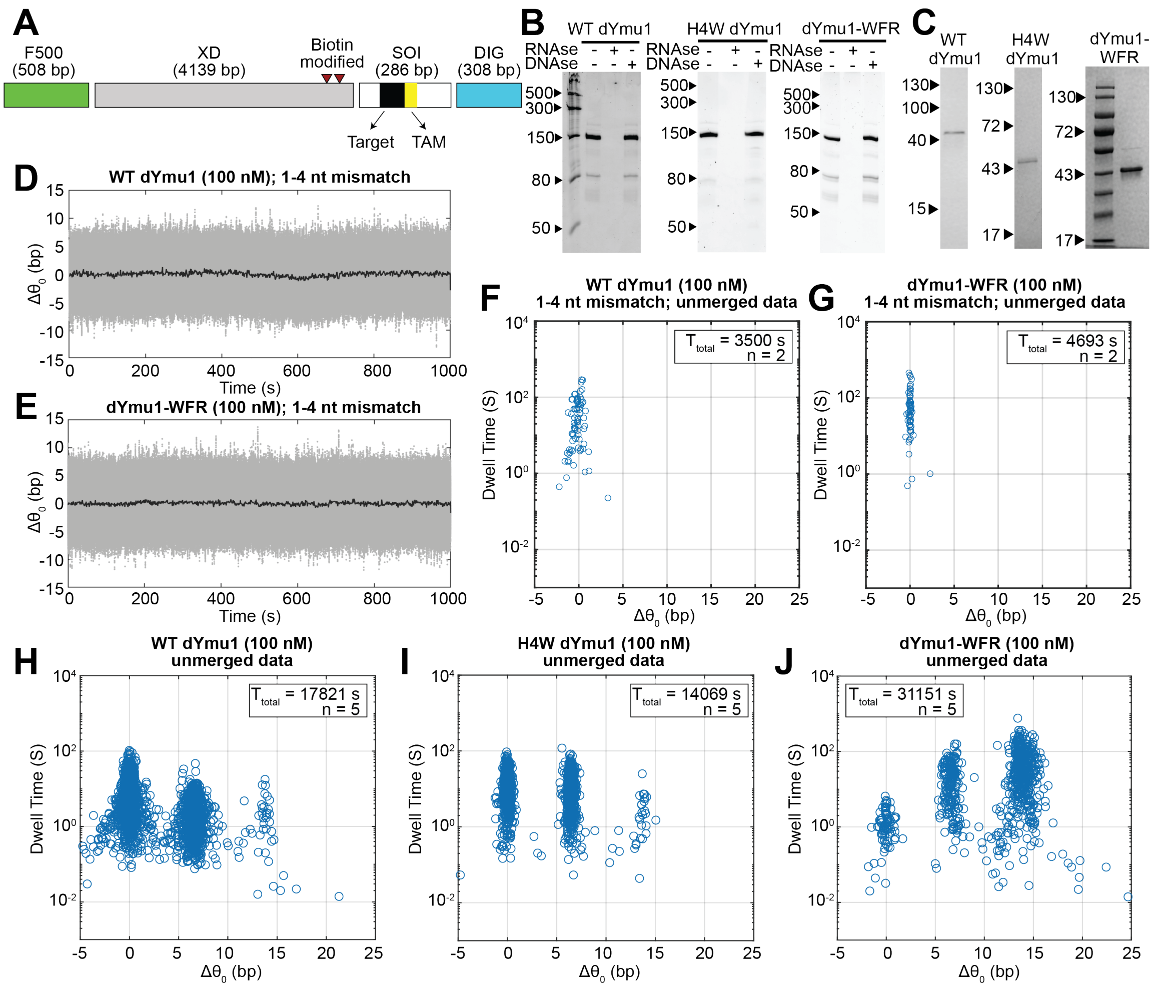

Fig. S4. Controls and validation for equilibrium AuRBT experiments (related to Fig. 4).

(A) Schematic of DNA tether used in AuRBT. Tethers were assembled by ligating four PCR-generated fragments (Table S14). The F500 and DIG segments contained fluorescein- and digoxigenin-modified dUTPs for bead and coverslip attachment, respectively. The transducer segment (XD) carried two biotin modifications for rotor-bead linkage. The sequence-of-interest (SOI) included a TTGAT TAM followed by the Target 1 sequence for dYmu1 TnpB binding (Table S15). The full tether sequence is provided in Table S16. **(B)** Denaturing PAGE analysis of the reRNA homogeneity for WT dYmu1, H4W dYmu1, and dYmu1-WFR TnpB samples used in torsionally-relaxed equilibrium AuRBT experiments. Gels are shown under untreated, RNase-treated, and DNase-treated conditions. **(C)** SDS-PAGE analysis of the TnpB protein homogeneity for WT dYmu1, H4W dYmu1, and dYmu1-WFR samples used in torsionally-relaxed equilibrium AuRBT experiments. **(D-E)** Representative trajectories of $\Delta\theta_0$ (bp) over time (sec) using tethers containing DNA-RNA mismatches introduced into nt 1-4 of the target DNA sequence. **(D)** WT dYmu1 and **(E)** dYmu1-WFR. Low-pass-filtered traces (1 Hz) are shown in black. **(F-G)** Scatter plots of dwell times (sec) versus $\Delta\theta_0$ (bp) for all unmerged Steppi-assigned dwells for experiments with 1-4 nt DNA-RNA mismatches. **(F)** WT dYmu1 and **(G)** dYmu1-WFR. The total collection time (T_{total}) and the number of DNA tethers analyzed (n) are reported in the figure legend. *R*-loop formation events are not

observed, consistent with a requirement for initial seed matching. **(H-J)** Scatter plots of state dwell times (sec) versus $\Delta\theta_0$ (bp) for all unmerged Steppi-assigned states of **(H)** WT dYmu1, **(I)** H4W dYmu1, and **(J)** dYmu1-WFR. The total collection time (T_{total}) and the number of DNA tethers analyzed (n) are reported in the figure legend. Complete AuRBT trace statistics, including the numbers of detected transitions, are summarized in Table S3.

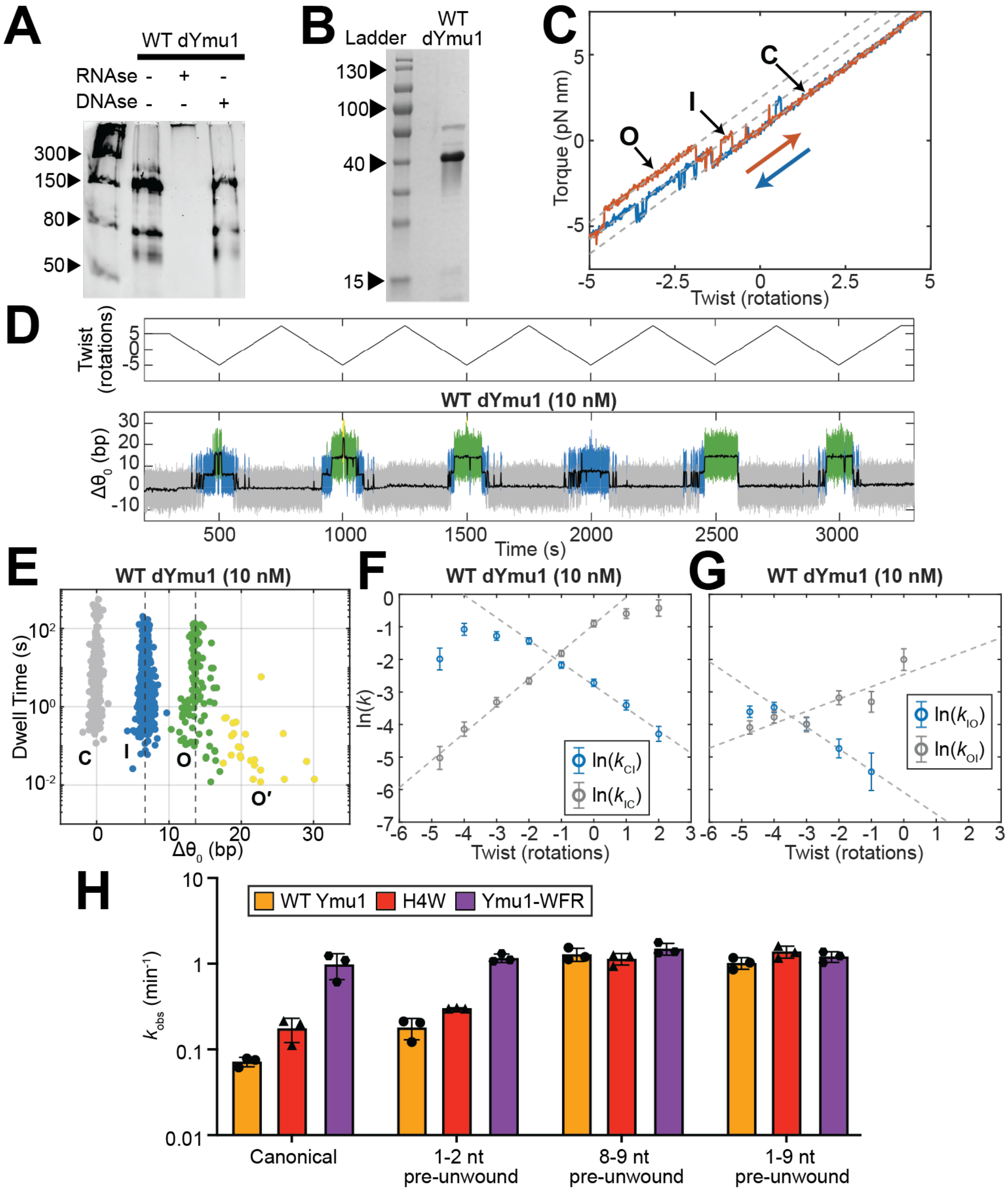

Fig. S5. Controls and additional analyses for torque-driven DNA-unwinding experiments (related to Fig. 5) (A) Denaturing PAGE analysis of the reRNA homogeneity for the WT dYmu1 sample used in torque-driven AuRBT experiments. RNA gels are shown under untreated, RNase-treated, and DNase-treated conditions. **(B)** SDS-PAGE of purified dYmu1 TnpB protein used in the torque-driven AuRBT experiments. The WT dYmu1 RNP sample in Fig. 5 and Fig. S5 was prepared independently from that used in equilibrium AuRBT assays. **(C)** Torque vs. twist for a twist ramping cycle in the presence of WT dYmu1. Blue (unwinding) and orange (rewinding) arrows indicate the direction of ramping. **(D)** Representative twist ramping trace (a portion of this trace is shown in Fig. 5B). (Top) Imposed twist

(rotations). (Bottom) $\Delta\theta_0$ (expressed in units of base-pairs unwound assuming changes arise from local DNA unwinding of B-DNA). Transitions between distinct states are scored using automated change-point detection followed by merging to prevent overscoring[1,2]. States are color-coded as closed (C, gray), intermediate (**I**, blue), open (O, green), and extended open (O′, yellow). Low-pass-filtered traces (1 Hz) are shown in black. **(E)** Scatter plot of dwell times (sec) versus $\Delta\theta_0$ (bp) after merging (Methods) for torque-driven measurements of WT dYmu1. Corresponding unmerged dwells are shown in Fig. 5C. Distinct unwinding states include closed (C, gray), intermediate (**I**, blue), open (O, green), and an extended open (O′, yellow). **(F-G)** Plots of ln(*k*) versus imposed twist (rot), for forward and reverse transition rates k_{ij} in s^{-1} of **(F)** C↔**I** and **(G)** **I**↔O transitions for WT dYmu1. Data points are listed in Table S4 and linear fit parameters are summarized in Table S5. AuRBT trace statistics, including the number of tethers analyzed, total tracking time, and number of detected transitions, are summarized in Table S6. **(H)** Pre-unwound DNA cleavage assays showing apparent cleavage rates (k_{obs}) as a function of initial duplex opening of WT, H4W, and Ymu1-WFR. Shown are TS cleavage. NTS cleavage profiles are provided in Fig. 5H.

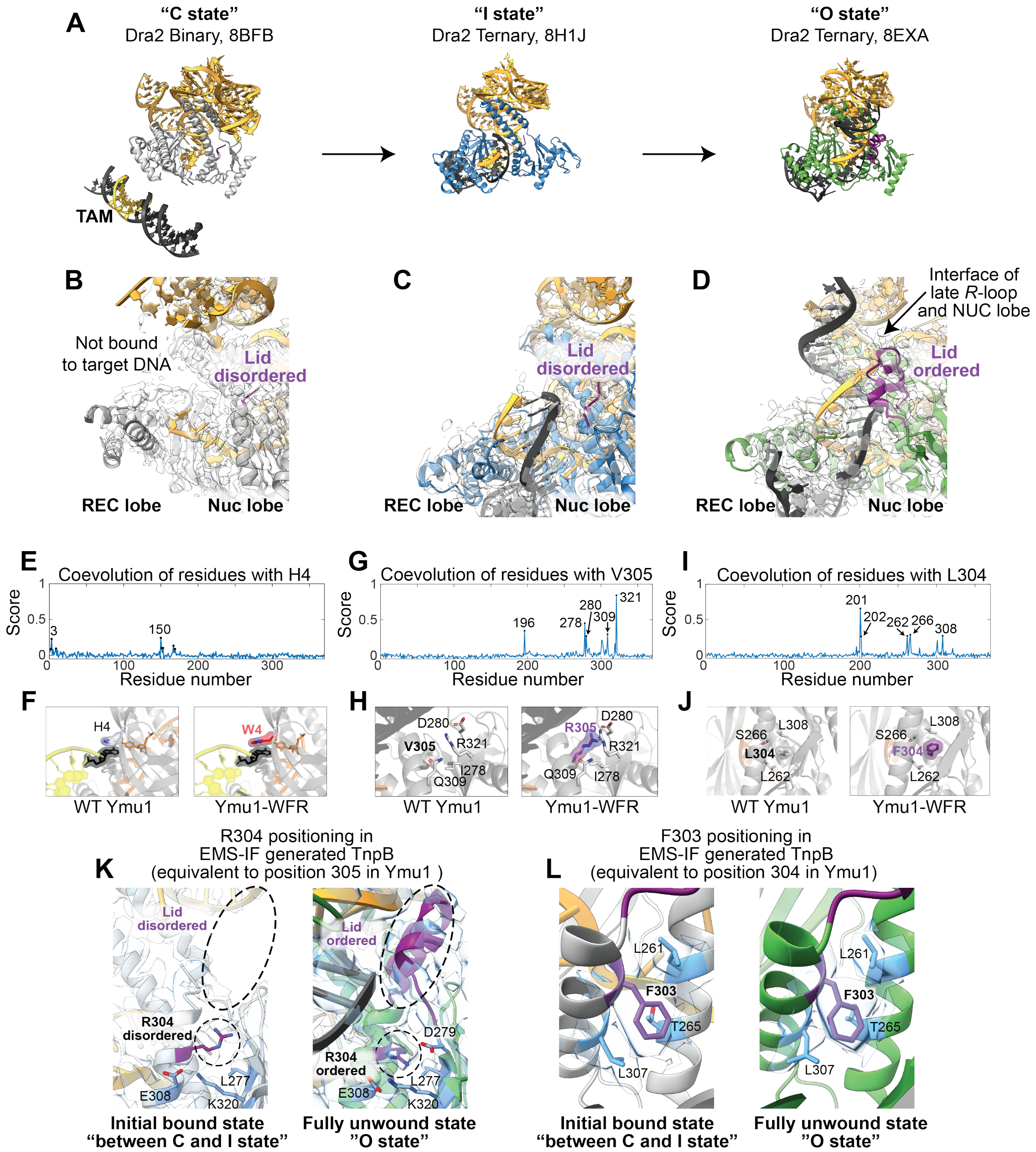

Fig. S6. Analyses supporting mutational effects at positions 4, 305, and 304 (related to Fig. 6).
(A) Comparison of cryo-EM structures of Dra2 TnpB in distinct conformational states. The closed ("C") state is defined by intact duplex DNA and corresponds to a DNA-free or binary-like protein complex conformation in solution. The Dra2 binary complex lacking target DNA[3] (PDB: 8BFB) is therefore shown as a reference for the protein conformation associated with the C state. The ternary state reported by Nakagawa et al.[4] (PDB: 8H1J) is proposed to correspond to the intermediate ("I") state, and the ternary state reported by Sasnauskas et al.[3] (PDB: 8EXA) is proposed to correspond to the fully-formed open ("O") state. **(B-D)** Experimentally resolved heteroduplex and lid domain structures across conformational states of Dra2 TnpB. **(B)** In 8BFB ("C state"), the lid domain is disordered and DNA is not unwound. **(C)** In

8H1J ("I state"), the lid remains mostly unstructured, possibly allowing transient interactions with a partially formed DNA heteroduplex, which mostly binds the REC lobe. 6-8 bp of heteroduplex are most strongly resolved in the density; 8 bp are shown in the figure, fewer than the 12 bp included in the 8H1J model[4]. **(D)** In 8EXA ("O state"), the lid domain becomes ordered and facilitates binding of TnpB with the RNA-DNA heteroduplex along its whole length, consistent with the fully unwound O state. The heteroduplex is stabilized by both the REC and the NUC lobes. **(E)** Co-evolution profile for residue 4 with other protein residues across ~4,500 non-redundant TnpB homologs. **(F)** AF3 ternary-complex model of Ymu1 TnpB highlighting residue 4 in the WED domain. The H4W substitution is positioned adjacent to the first RNA-DNA base pair of the heteroduplex. **(G)** Co-evolution profile for residue 305. **(H)** AF3 ternary-complex model highlighting residue 305 within the lid-adjacent helix, proximal to a network of residues in the catalytic RuvC domain. **(I)** Co-evolution profile for residue 304. **(J)** AF3 ternary-complex model highlighting residue 304, located within a hydrophobic interface between the lid-adjacent and bridge helices. **(K-L)** Local structural environments of residues in cryo-EM structures of an ESM inverse-folding (ESM-IF) generated TnpB[5] homologous to the Ymu1 WFR substitutions. **(K)** Residue R304 in the ESM-IF-generated TnpB (homologous to Ymu1 V305R substitution) does not form cryo-EM density-supported contacts in the initial bound state, whereas it is associated with a strong density in the fully unwound state. **(L)** Residue F303 in the ESM-IF-generated TnpB (homologous to Ymu1 substitution L304F) is structured in both early and late *R*-loop states. For each panel, the left view shows an initial bound state preceding the *R*-loop formation, and the right view shows the fully unwound state. Density and model views illustrate state-dependent ordering of the lid region and associated residues. Cryo-EM maps in **(B-D)** were contoured at 7.0, 0.5, 8.0, respectively, whereas **(K-L)** were contoured at 0.1 (dataset-dependent map units).

Supplemental Discussion

Discussion S1. Phylogenetic and structural analysis of Ymu1 TnpB mutations

To understand how mutations in Ymu1-WFR reshape the DNA unwinding landscape, we combined sequence co-evolution analysis[6] of ~4,500 non-redundant TnpB homologs, AlphaFold3 (AF3) modeling of the Ymu1-reRNA-DNA ternary complex, and comparison with available cryo-EM structures of related TnpB homologs[3-5] (see Methods). Together, these analyses suggest two classes of effects: stabilization of early RNA-DNA engagement and modulation of protein conformational transitions that favor later, more extensively unwound states.

<u>Proposed structural correspondence of C, I, and O states</u>

We propose a structural framework relating cryo-EM conformations of a related ortholog (Dra2) to the unwinding intermediates resolved by AuRBT. The Dra2 protein-RNA binary complex[3] is most relevant to the closed (C) state (Fig. S6A-B). The Dra2 ternary structure reported by Nakagawa et al.[4] exhibits cryo-EM density consistent with limited ordering of the TAM-distal RNA-DNA heteroduplex, and the strongly resolved proximal heteroduplex principally engages the REC lobe (Fig. S6A, S6C). We propose that the partially unwound intermediate (I) state observed in our experiments, in which only a short RNA-DNA heteroduplex is formed, may adopt a protein-RNA-DNA architecture resembling this structure. By contrast, the ternary structure reported by Sasnauskas et al.[3], which resolves ~15 bp of ordered heteroduplex, may correspond to the fully unwound open (O) state (Fig. S6A, S6D). In this putative O-state architecture, the extended RNA-DNA heteroduplex engages both the REC and NUC lobes, including the bridge helix and lid domain (Fig. S6D). Across these structures, the lid domain appears disordered in the C state and becomes progressively ordered. We propose the lid may have transient interactions with the partially formed RNA-DNA heteroduplex in the I state, and then becomes well-ordered and engages the heteroduplex in the fully unwound O state, suggesting coupling between lid ordering and late-stage *R*-loop formation as observed in related Cas12 enzymes[7-9]. AF3 modeling of the Ymu1 ternary complex converges on a similar fully unwound O-state architecture (Methods), providing a structural reference for interpreting mutational effects discussed shortly.

<u>Proposed role of H4W in initiation of unwinding</u>

While sequence co-evolution analysis revealed little evidence of coupling between H4 and other amino acids in TnpB (Fig. S6E), AF3 modeling places H4 adjacent to the first RNA-DNA base pair of the heteroduplex (Fig. S6F). Substitution of His with Trp may introduce a larger aromatic surface to enhance π-stacking at the heteroduplex terminus, stabilizing initial heteroduplex formation. Together with our AuRBT data showing preferential stabilization of the I state by H4W, and prior reports of activity-enhancing substitutions at homologous positions in TnpB and Cas12f

enzymes[10,11], these observations support a model in which H4W enhances unwinding by stabilizing early RNA-DNA engagement.

<u>Proposed role of L304F and V305R in post-initiation unwinding</u>

Co-conservation analysis identifies several evolutionarily coupled residues surrounding V305 (I278, D280, Q309, and R321) (Fig. S6G), consistent with the AF3 model in which V305R is immediately adjacent to this network (Fig. S6H). More specifically, the AF3-predicted arginine 305 residue in the lid-adjacent helix is oriented to form an interaction with D280 on the counterpart RuvC β-strand, with I278, Q309, and R321 forming additional contacts, suggesting a molecular mechanism for lid domain stabilization in the ternary conformation. Co-evolution analysis of L304 likewise identifies a hydrophobic cluster (L262, S266, L308), consistent with AF3 modeling, which places L304 in a tightly packed hydrophobic pocket formed by these residues from the lid-adjacent helix and the bridge helix (Fig. S6I-J). Substitution with a bulkier aromatic side chain (L304F) could provide improved packing within this pocket. Consistent with this interpretation, deep mutational scanning of Dra2 TnpB indicates that the native Phe is preferred over Leu at the position homologous to Ymu1 L304[11]. Together, these structural and covariation analyses support a model in which L304F and V305R strengthen interactions within the lid-proximal region of the RuvC domain, thereby stabilizing the I and O unwound states and complementing the effect of H4W, consistent with our AuRBT experiment (Fig. 4). The greater stabilization conferred by WFR in the O state compared with the I state (energy minima in Fig. 4J) could be explained by increased lid ordering and larger contributions to stability for lid-adjacent residues in the O state. In the I state the shorter heteroduplex adopts a distinct REC-bound confirmation that may support only transient interactions with the lid domain, whereas the lid is ordered and engages the fully formed heteroduplex in the O state.

Recent cryo-EM structures of an artificial TnpB designed using evolutionary information and ESM inverse folding (ESM-IF), in which F303 and R304 occupy positions homologous to Ymu1 L304 and V305 in a similar residues context, provide additional structural explanation to the proposed stabilization mechanisms[5]. Across reported conformations, both the lid domain and the Ymu1's V305-homologous residue R304 are unresolved in the initial TAM-bound structure (which may correspond to a state intermediate between C and I) but become ordered in the fully unwound state (O) (Fig. S6K). Based on this experimental observation, we suggest that Ymu1's V305R similarly supports formation of the RuvC and lid domain conformations in the late unwound states (Fig. S6K). In contrast, F303 is structured in both early and late states (Fig. S6L), and may contribute to overall RuvC fold stabilization across the unwinding pathway.

Supplemental Tables

Table S1. Target site sequences and amplicon-sequencing primers used in plant genome-editing experiments (related to Fig. 1 and Methods)

Gene ID	Target site	Target site sequence (5′-3′)	Forward primer sequence (5′-3′)	Reverse primer sequence (5′-3′)
AT4G14210	AtPDS3_g2	aaggcaaattcgccgc	ACACTCTTTCCCTACACGACGCTCTTCCGATCTgaagcagttgtgagttaagttggaga	GTGACTGGAGTTCAGACGTGTGCTCTTCCGATCTttgtcttaagcgcttgagaagtgg
AT4G18480	AtCHLI1_g4	CTGTTACCTGAGATTA	ACACTCTTTCCCTACACGACGCTCTTCCGATCTGTGGTGTTATGATTATGGGAGATAGAG	GTGACTGGAGTTCAGACGTGTGCTCTTCCGATCTTCGCAATAACAGGAACTTGCTC
AT4G18480	AtCHLI1_g6	GAAGTTAATCTCTTGG	ACACTCTTTCCCTACACGACGCTCTTCCGATCTAAGCCTTTGAGCCTGGTTTG	GTGACTGGAGTTCAGACGTGTGCTCTTCCGATCTCGGGTGAGAAATCGAAATCCC
AT4G18480	AtCHLI1_g9	CGGTTTGGTATGCATG	ACACTCTTTCCCTACACGACGCTCTTCCGATCTGCGAGGTTTATCTTGATCGGTTC	GTGACTGGAGTTCAGACGTGTGCTCTTCCGATCTTCACGGAAATCCTTTGGGTTACTA
AT5G45930	AtCHLI2_g1	CTCTGTAGCGACATTC	ACACTCTTTCCCTACACGACGCTCTTCCGATCTtgcagAGAAACTTTCTGGAAGAATCCA	GTGACTGGAGTTCAGACGTGTGCTCTTCCGATCTtcattccaaaggttcaagcttttaatc
AT5G45930	AtCHLI2_g3	TTACCAGTTCCTCTAT	ACACTCTTTCCCTACACGACGCTCTTCCGATCTGGACAAGATGAGATGAAGCTATGCCTT	GTGACTGGAGTTCAGACGTGTGCTCTTCCGATCTCTCGGGTCTGAGTTATACGGATCA
AT5G45930	AtCHLI2_g8	GAAGTTAATCTTTTGG	ACACTCTTTCCCTACACGACGCTCTTCCGATCTAGGCGTTTGAGCCTGGACTACTA	GTGACTGGAGTTCAGACGTGTGCTCTTCCGATCTAGCAGGATGAGAAATCGATATCCCTTC
AT5G45930	AtCHLI2_g10	TCAGCTGCATCTGGTT	ACACTCTTTCCCTACACGACGCTCTTCCGATCTTAGCTAAAGCTAATAGAGGGATTCTTTATGTTGAT	GTGACTGGAGTTCAGACGTGTGCTCTTCCGATCTGTGGTCTAAGCTCTCCTTCTTCAGGA
AT2G46410	AtCPC_g1	cggtttgagtctgatt	ACACTCTTTCCCTACACGACGCTCTTCCGATCTCCAAGGCTTCTTGTTCCGAAG	GTGACTGGAGTTCAGACGTGTGCTCTTCCGATCTCCAACGAGTTTATACATCCGAGAA

AT2G46410	AtCPC_g5	tacagcatgtttgtat	ACACTCTTTCCCTAC ACGACGCTCTTCCGA TCTtgattctttagcaaaacat attcttaatttatgtca	GTGACTGGAGTTCAG ACGTGTGCTCTTCCG ATCTccgataaaaaccgcat aaagtttgt
AT4G14210	AtPDS3_g5	tacccatcctaaagta	ACACTCTTTCCCTAC ACGACGCTCTTCCGA TCTgacgtcaggaagaacat ggtcatttg	GTGACTGGAGTTCAG ACGTGTGCTCTTCCG ATCTagaacatttcagcgct aatgctacaa
AT4G14210	AtPDS3_g10	taacttgtactacctc	ACACTCTTTCCCTAC ACGACGCTCTTCCGA TCTtgttgcagcttatgacaca caataag	GTGACTGGAGTTCAG ACGTGTGCTCTTCCG ATCTgaatctttatctcactgg caatcata
AT4G14210	AtPDS3_g12	gcgttggagcatataa	ACACTCTTTCCCTAC ACGACGCTCTTCCGA TCTgaaccgacccgagaag agatttg	GTGACTGGAGTTCAG ACGTGTGCTCTTCCG ATCTtggaatacacacatttg tacaacca
AT5G53200	AtTRY_g3	AGCAGGAAGAGTTCCT	ACACTCTTTCCCTAC ACGACGCTCTTCCGA TCTtttattatgaaaatataaat gctaatgcttgggat	GTGACTGGAGTTCAG ACGTGTGCTCTTCCG ATCTTGGCGTCGTTT ATCAGCAAAG

Table S2. Transition rate constants from equilibrium AuRBT assays with fully matched target sequences (related to Fig. 4 and Fig. S4)

RNP	Conc	$k_{C\to I}$ (s^{-1})	$k_{C\to I}$ error (s^{-1})	$k_{I\to C}$ (s^{-1})	$k_{I\to C}$ error (s^{-1})	$k_{I\to O}$ (s^{-1})	$k_{I\to O}$ error (s^{-1})	$k_{O\to I}$ (s^{-1})	$k_{O\to I}$ error (s^{-1})
WT dYmu1	100 nM	0.084199	0.0024522	0.31662	0.0092328	0.010231	0.0016597	0.36536	0.05927
H4W dYmu1	100 nM	0.063184	0.0029022	0.073818	0.0033763	0.0043241	0.00081717	0.29539	0.056847
dYmu1-WFR	100 nM	0.62308	0.071006	0.01729	0.0019577	0.012856	0.0016881	0.002112	0.00028222

Table S3. AuRBT trace statistics for equilibrium AuRBT assays (related to Fig. 4, Fig. S4, and Methods)

Condition	Tethers	Unique Chambers	Time Tracked (s)	C → I	I → C	I → O	O → I	C → O	O → C
WT dYmu1 (100nM)	5	3	17821	1179	1176	38	38	2	3
H4W dYmu1 (100nM)	5	5	14069	474	478	28	27	1	2
dYmu1-WFR (100nM)	5	4	31151	77	78	58	56	2	1

WT dYmu1 (100nM) 1-4 nt mismatch	2	1	3500	0	0	0	0	0	0
dYmu1-WFR (100 nM) 1-4 nt mismatch	2	1	4693	0	0	0	0	0	0

Column "i → j" gives number of transitions observed from state i to state j.

Table S4. Transition rate constants of WT dYmu1 for non-equilibrium, torsion-driven AuRBT assays with fully matched target sequences (related to Fig. 5 and Fig. S5)

Twist	$k_{C\to I}$ [s^{-1}]	$k_{C\to I}$ error [s^{-1}]	$k_{I\to C}$ [s^{-1}]	$k_{I\to C}$ error [s^{-1}]	$k_{I\to O}$ [s^{-1}]	$k_{I\to O}$ error [s^{-1}]	$k_{O\to I}$ [s^{-1}]	$k_{O\to I}$ error [s^{-1}]
-4.75	0.1371	0.0457	0.0066	0.0023	0.0270	0.0047	0.0167	0.0034
-4	0.3410	0.0633	0.0159	0.0035	0.0310	0.0048	0.0230	0.0042
-3	0.2784	0.0366	0.0363	0.0052	0.0185	0.0037	0.0184	0.0042
-2	0.2387	0.0231	0.0699	0.0071	0.0087	0.0025	0.0415	0.0088
-1	0.1139	0.0112	0.1624	0.0152	0.0043	0.0025	0.0367	0.0116
0	0.0657	0.0073	0.4068	0.0413	NA	NA	0.1347	0.0449
1	0.0331	0.0049	0.5525	0.0815	NA	NA	NA	NA
2	0.0137	0.0031	0.6544	0.1636	NA	NA	NA	NA
3	0.0021	0.0012	0.9507	0.4753	NA	NA	NA	NA
4	NA	NA	NA	NA	NA	NA	NA	NA

Table S5. Parameters from linear fits of ln(K_{ij}) and ln(k_{ij}) vs imposed twist (related to Fig. 5 and Fig. S5)

Transition	$\Delta\theta_{ij}$ (bp)	$\Delta G_{ij}(0)$ (k_BT)	ΔG_{ij}(-5) (k_BT)	$\Delta\theta^{\ddagger}{}_{ij}$ (bp)	$ln(k_{ij}(0))$	$\Delta\theta^{\ddagger}{}_{ji}$ (bp)	$ln(k_{ji}(0))$
C↔I	5.3	1.6	-3.9	3.2	-2.8	-3.9	-0.9
I↔O	3.6	2.8	-1.2	3.1	-6.1	-1.7	-2.5

$\Delta\theta_{ij}$, reported in number of base pairs unwound, gives the predicted difference in equilibrium twist between states i and j based on the slope of the $ln(K_{ij})$ vs twist plot. $\Delta G_{ij}(0)$ and ΔG_{ij}(-5) give the predicted free energy difference between the states at 0 and -5 twist, respectively. Linear fits calculated from equally-weighted $ln(K_{ij})$ data points.

$\Delta\theta^{\ddagger}_{ij}$, reported in the number of base pairs unwound, gives the predicted location of the transition state for the i→j transition in relation to the predicted equilibrium twist for state i. $ln(k_{ij}(0))$ gives the natural log of the predicted transition rate (in s^{-1}) from state i to state j at 0 twist. Linear fits calculated from equally-weighted data points selected from a linear portion of $ln(k_{ij})$ data.

Table S6. AuRBT trace statistics for non-equilibrium torque-driven assays (related to Fig. 5, Fig. S5, and Methods)

Condition	Tethers	Unique Chambers	Cycles
WT dYmu1 (10nM)	13	13	68

Twist interval	C → I	I → C	I → O	O → I
[-5, -4.5]	9	8	33	24
[-4.5, -3.5]	29	21	41	30
[-3.5, -2.5]	58	49	25	19
[-2.5, -1.5]	107	96	12	22
[-1.5, -0.5]	104	114	3	10
[-0.5, 0.5]	82	97	1	9
[0.5, 1.5]	45	46	0	0
[1.5, 2.5]	19	16	0	0
[2.5, 3.5]	3	4	0	0
[3.5, 4.5]	2	2	0	0
[4.5, 5.5]	0	0	0	0
[5.5, 6.5]	0	0	0	0
[6.5, 7.5]	0	0	0	0

Column “i → j” gives number of transitions observed from state i to state j.

Table S7. Rate constants (k_{obs}) for DNA cleavage assay (n=3) (related to Fig. 3, Fig. 5 and Fig. S3, Fig. S5)

Protein	Target	Strand	Mean (min^{-1})	SD (min^{-1})
**WT Ymu1**	1	NTS	0.0676	0.0033

		TS	0.0717	0.0090
	2	NTS	0.0152	0.0019
		TS	0.0179	0.0019
**H4W**	1	NTS	0.168	0.039
		TS	0.176	0.055
**V305R**	1	NTS	0.198	0.025
		TS	0.179	0.023
**Ymu1-WFR**	1	NTS	2.00	0.74
		TS	0.980	0.328
	2	NTS	0.0793	0.0038
		TS	0.0380	0.0032
**L304F**	1	NTS	0.0692	0.0043
		TS	0.0737	0.0023

Table S8. Rate constants (k_{obs}) for pre-unwound DNA cleavage assay (n=3) (related to Fig. 5 and Fig. S5)

Strand	DNA target	WT Ymu1 mean (min^{-1})	WT Ymu1 SD (min^{-1})	H4W mean (min^{-1})	H4W SD (min^{-1})	Ymu1-WFR mean (min^{-1})	Ymu1-WFR SD (min^{-1})
NTS	1-2 nt pre-unwound substrate	0.162	0.040	0.217	0.060	1.42	0.16
	8-9 nt pre-unwound substrate	1.77	0.13	1.57	0.12	2.17	0.18
	1-9 nt pre-unwound substrate	1.51	0.20	2.17	0.65	2.44	0.33
TS	1-2 nt pre-unwound substrate	0.180	0.050	0.301	0.004	1.16	0.13
	8-9 nt pre-unwound substrate	1.29	0.22	1.14	0.18	1.49	0.24
	1-9 nt pre-unwound substrate	1.02	0.16	1.38	0.22	1.21	0.17

The k_{obs} value for the canonical substrate is shown in Table S7.

Table S9. Plasmid vectors used in this study (related to Methods)

Internal ID	System	Purpose
pZZ09	*E.coli*	Bacterial expression of Ymu1 protein
pHS355	*E.coli*	Bacterial expression of Dra2 protein
pZZ049	*E.coli*	Bacterial expression of Tel2 protein
pZZ034	*E.coli*	Bacterial expression of Tfu1 protein
pZZ053	*E.coli*	Bacterial expression of Ec41 protein
pZZ031	*E.coli*	Bacterial expression of dYmu1 protein
pZZ069	*E.coli*	Bacterial expression of V305R Ymu1 protein
pZZ080	*E.coli*	Bacterial expression of H4W Ymu1 protein
pZZ087	*E.coli*	Bacterial expression of H4W dYmu1 protein
pHS749	*E.coli*	Bacterial expression of Ymu1-WFR protein
pHS750	*E.coli*	Bacterial expression of dYmu1-WFR protein
pZZ089	*E.coli*	Bacterial expression of L304F Ymu1 protein

pHS607	*E.coli*	Bacterial expression of Ymu1 200-nt reRNA for target 1
pHS610	*E.coli*	Bacterial expression of Ymu1 127-nt reRNA for target 1
pHS612	*E.coli*	Bacterial expression of Ymu1 short reRNA for target 1
pZZ062	*E.coli*	Bacterial expression of Ymu1 short reRNA for target 2
pZZ064	*E.coli*	Bacterial expression of Ymu1 127-nt reRNA for target 2
pZZ013	*E.coli*	Bacterial expression of Dra2 200-nt reRNA for target 1
pZZ052	*E.coli*	Bacterial expression of Tel2 200-nt reRNA for target 1
pZZ035	*E.coli*	Bacterial expression of Tfu1 200-nt reRNA for target 1
pZZ054	*E.coli*	Bacterial expression for Ec41 200-nt reRNA for target 1
pHS516	*E.coli*	Bacterial TAM assay vector encoding WT Ymu1 and WT reRNA expressed as a single transcript, with a 16-nt guide flanked by HDV
pHS751	*E.coli*	Bacterial TAM assay vector encoding Ymu1-WFR and WT reRNA expressed as a single transcript, with a 16-nt guide flanked by HDV
pTW2036	*Arabidopsis*	WT Ymu1 and WT reRNA with 14 nt spacer length targeting AtPDS3_g2
pTW2035	*Arabidopsis*	WT Ymu1 and WT reRNA with 15 nt spacer length targeting AtPDS3_g2
pMK061	*Arabidopsis*	WT Ymu1 and WT reRNA with 16 nt spacer length targeting AtPDS3_g2
pTW2034	*Arabidopsis*	WT Ymu1 and WT reRNA with 17 nt spacer length targeting AtPDS3_g2
pTW2033	*Arabidopsis*	WT Ymu1 and WT reRNA with 18 nt spacer length targeting AtPDS3_g2
pTW2032	*Arabidopsis*	WT Ymu1 and WT reRNA with 19 nt spacer length targeting AtPDS3_g2
pTW2031	*Arabidopsis*	WT Ymu1 and WT reRNA with 20 nt spacer length targeting AtPDS3_g2
pTW2314	*Arabidopsis*	G285A Ymu1 variant with short reRNA targeting AtPDS3_g2
pTW2315	*Arabidopsis*	G285I Ymu1 variant with short reRNA targeting AtPDS3_g2
pTW2317	*Arabidopsis*	G285V Ymu1 variant with short reRNA targeting AtPDS3_g2
pTW2321	*Arabidopsis*	H4W-L304F Ymu1 variant with short reRNA targeting AtPDS3_g2
pTW2322	*Arabidopsis*	H4W-L304F-V305R Ymu1 variant with short reRNA targeting AtPDS3_g2
pTW2397	*Arabidopsis*	H4W-V305R Ymu1 variant with short reRNA targeting AtPDS3_g2
pTW2335	*Arabidopsis*	L304F-V305R Ymu1 variant with short reRNA targeting AtPDS3_g2

pTW2145	*Arabidopsis*	WT Ymu1 with WT reRNA targeting AtCHLI1_g4
pTW2149	*Arabidopsis*	WT Ymu1 with WT reRNA targeting AtCHLI1_g6
pTW2125	*Arabidopsis*	WT Ymu1 with WT reRNA targeting AtCHLI1_g9
pTW2126	*Arabidopsis*	WT Ymu1 with WT reRNA targeting AtCHLI2_g1
pTW2128	*Arabidopsis*	WT Ymu1 with WT reRNA targeting AtCHLI2_g3
pTW2147	*Arabidopsis*	WT Ymu1 with WT reRNA targeting AtCHLI2_g8
pTW2134	*Arabidopsis*	WT Ymu1 with WT reRNA targeting AtCHLI2_g10
pTW2198	*Arabidopsis*	WT Ymu1 with WT reRNA targeting AtCPC_g1
pTW2202	*Arabidopsis*	WT Ymu1 with WT reRNA targeting AtCPC_g5
pMK064	*Arabidopsis*	WT Ymu1 with WT reRNA targeting AtPDS3_g5
pMK068	*Arabidopsis*	WT Ymu1 with WT reRNA targeting AtPDS3_g10
pMK070	*Arabidopsis*	WT Ymu1 with WT reRNA targeting AtPDS3_g12
pTW2196	*Arabidopsis*	WT Ymu1 with WT reRNA targeting AtTRY_g3
pTW2541	*Arabidopsis*	H4W-L304F-V305R Ymu1 with WT reRNA targeting AtPDS3_g2
pTW2532	*Arabidopsis*	H4W-L304F-V305R Ymu1 with WT reRNA targeting AtCHLI1_g4
pTW2533	*Arabidopsis*	H4W-L304F-V305R Ymu1 with WT reRNA targeting AtCHLI1_g6
pTW2534	*Arabidopsis*	H4W-L304F-V305R Ymu1 with WT reRNA targeting AtCHLI1_g9
pTW2536	*Arabidopsis*	H4W-L304F-V305R Ymu1 with WT reRNA targeting AtCHLI2_g8
pTW2537	*Arabidopsis*	H4W-L304F-V305R Ymu1 with WT reRNA targeting AtCHLI2_g10
pTW2542	*Arabidopsis*	H4W-L304F-V305R Ymu1 with WT reRNA targeting AtPDS3_g5
pTW2543	*Arabidopsis*	H4W-L304F-V305R Ymu1 with WT reRNA targeting AtPDS3_g10
pTW2471	*Arabidopsis*	H4W Ymu1with WT reRNA targeting AtPDS3_g2
pTW2503	*Arabidopsis*	H4W-L304F-V305R Ymu1 with WT reRNA ccdb gRNA cloning vector
pTW2453	*Arabidopsis*	H4W Ymu1 with WT reRNA ccdb gRNA cloning vector
pMK525	*Arabidopsis*	ccdb TnpB cloning vector
pMK025	*Arabidopsis*	WT Ymu1 with WT reRNA ccdb gRNA cloning vector
pKV100	*Arabidopsis*	H4Y Ymu1 variant with short reRNA targeting AtPDS3_g2
pKV101	*Arabidopsis*	H4F Ymu1 variant with short reRNA targeting AtPDS3_g2
pKV102	*Arabidopsis*	H4W Ymu1 variant with short reRNA targeting AtPDS3_g2
pKV106	*Arabidopsis*	K229A Ymu1 variant with short reRNA targeting AtPDS3_g2

pKV105	*Arabidopsis*	K229E Ymu1 variant with short reRNA targeting AtPDS3_g2
pKV111	*Arabidopsis*	K229I Ymu1 variant with short reRNA targeting AtPDS3_g2
pKV112	*Arabidopsis*	K229L Ymu1 variant with short reRNA targeting AtPDS3_g2
pKV104	*Arabidopsis*	K229Q Ymu1 variant with short reRNA targeting AtPDS3_g2
pKV103	*Arabidopsis*	K229R Ymu1 variant with short reRNA targeting AtPDS3_g2
pKV113	*Arabidopsis*	K229V Ymu1 variant with short reRNA targeting AtPDS3_g2
pKV110	*Arabidopsis*	R230A Ymu1 variant with short reRNA targeting AtPDS3_g2
pKV109	*Arabidopsis*	R230E Ymu1 variant with short reRNA targeting AtPDS3_g2
pKV114	*Arabidopsis*	R230I Ymu1 variant with short reRNA targeting AtPDS3_g2
pKV107	*Arabidopsis*	R230K Ymu1 variant with short reRNA targeting AtPDS3_g2
pKV115	*Arabidopsis*	R230L Ymu1 variant with short reRNA targeting AtPDS3_g2
pKV108	*Arabidopsis*	R230Q Ymu1 variant with short reRNA targeting AtPDS3_g2
pKV116	*Arabidopsis*	R230V Ymu1 variant with short reRNA targeting AtPDS3_g2
pKV136	*Arabidopsis*	V283E Ymu1 variant with short reRNA targeting AtPDS3_g2
pKV138	*Arabidopsis*	V283I Ymu1 variant with short reRNA targeting AtPDS3_g2
pKV141	*Arabidopsis*	V283K Ymu1 variant with short reRNA targeting AtPDS3_g2
pKV140	*Arabidopsis*	V283L Ymu1 variant with short reRNA targeting AtPDS3_g2
pKV137	*Arabidopsis*	V283Q Ymu1 variant with short reRNA targeting AtPDS3_g2
pKV134	*Arabidopsis*	V283R Ymu1 variant with short reRNA targeting AtPDS3_g2
pKV135	*Arabidopsis*	V283Y Ymu1 variant with short reRNA targeting AtPDS3_g2
pKV139	*Arabidopsis*	M287R Ymu1 variant with short reRNA targeting AtPDS3_g2
pKV128	*Arabidopsis*	H290A Ymu1 variant with short reRNA targeting AtPDS3_g2
pKV129	*Arabidopsis*	H290E Ymu1 variant with short reRNA targeting AtPDS3_g2
pKV126	*Arabidopsis*	H290R Ymu1 variant with short reRNA targeting AtPDS3_g2
pKV127	*Arabidopsis*	H290Y Ymu1 variant with short reRNA targeting AtPDS3_g2
pKV132	*Arabidopsis*	A293E Ymu1 variant with short reRNA targeting AtPDS3_g2
pKV133	*Arabidopsis*	A293Q Ymu1 variant with short reRNA targeting AtPDS3_g2
pKV130	*Arabidopsis*	A293R Ymu1 variant with short reRNA targeting AtPDS3_g2
pKV131	*Arabidopsis*	A293Y Ymu1 variant with short reRNA targeting AtPDS3_g2
pKV120	*Arabidopsis*	S303F Ymu1 variant with short reRNA targeting AtPDS3_g2

pKV118	*Arabidopsis*	S303I Ymu1 variant with short reRNA targeting AtPDS3_g2
pKV124	*Arabidopsis*	S303K Ymu1 variant with short reRNA targeting AtPDS3_g2
pKV117	*Arabidopsis*	S303L Ymu1 variant with short reRNA targeting AtPDS3_g2
pKV125	*Arabidopsis*	S303M Ymu1 variant with short reRNA targeting AtPDS3_g2
pKV123	*Arabidopsis*	S303R Ymu1 variant with short reRNA targeting AtPDS3_g2
pKV119	*Arabidopsis*	S303V Ymu1 variant with short reRNA targeting AtPDS3_g2
pKV122	*Arabidopsis*	S303W Ymu1 variant with short reRNA targeting AtPDS3_g2
pKV121	*Arabidopsis*	S303Y Ymu1 variant with short reRNA targeting AtPDS3_g2
pKV160	*Arabidopsis*	L304F Ymu1 variant with short reRNA targeting AtPDS3_g2
pKV158	*Arabidopsis*	L304K Ymu1 variant with short reRNA targeting AtPDS3_g2
pKV161	*Arabidopsis*	V305R Ymu1 variant with short reRNA targeting AtPDS3_g2
pKV162	*Arabidopsis*	V305F Ymu1 variant with short reRNA targeting AtPDS3_g2
pKV161	*Arabidopsis*	V305R Ymu1 variant with short reRNA targeting AtPDS3_g2
pKV87	*Arabidopsis*	WT Ymu1 with short reRNA targeting AtPDS3_g2
pHS550	*Arabidopsis*	mRFP drop out vector with backbones for protoplast assay

Plasmids pHS516, pMK025, pMK061, pMK64, pMK68, pMK70 are from a previous study[12].

Table S10. Primers used for Gibson assembly-based site-directed mutagenesis of Ymu1 TnpB for bacterial expression (related to Methods)

Oligonucleotide	Internal ID	Sequence (5′-3′)
Reverse primer for amplifying Ymu1 backbone plasmid	oZZ080R	ttgacggcttgacggagtagcatagggtttgcag
Forward primer for amplifying Ymu1 backbone plasmid	oZZ081F	gcagggattctgcaaaccctatgctactccgtc
Forward primer to induce the E279A mutation in Ymu1	oZZ086F	AAACCACGATATCATCTGTATCgcgGACCTTAACGTTAAGGGCATGATGC
Reverse primer to induce the E279A mutation in Ymu1	oZZ087R	GCCCTTAACGTTAAGGTCcgcGATACAGATGATATCGTGGTTTTTGACTATCTCTGTAC
Reverse primer to induce the H4W mutation in Ymu1	oZZ227R	GTATTCATAGGCTTTccaCTGCAGCATTGCATTGGATTGG
Forward primer to induce the H4W mutation in Ymu1	oZZ228F	AATGCAATGCTGCAGtggAAAGCCTATGAATACCGTATCTATCCAGATAAGAAG
Forward primer to induce the both L304F/V305R mutations in	oZZ233F	TGGACGAGCttccgaTCGAAACTGCAGTACAAGGCTTC

Ymu1		
Reverse primer to induce the both L304F/V305R mutations in Ymu1	oZZ234R	CAGTTTCGAtcggaaGCTCGTCCATGATACATCAGAGATGC
Reverse primer to induce the L304F mutation in Ymu1	oZZ235R	CTGCAGTTTCGATACgaaGCTCGTCCATGATACATCAGAGATGC
Forward primer to induce the L304F mutation in Ymu1	oZZ236F	GTATCATGGACGAGCttcGTATCGAAACTGCAGTACAAGGCTTC
Forward primer to induce the V305R mutation in Ymu1	oZZ217F	TCATGGACGAGCCTTagaTCGAAACTGCAGTACAAGGCTTC
Reverse primer to induce the V305R mutation in Ymu1	oZZ220R	GTACTGCAGTTTCGAtctAAGGCTCGTCCATGATACATCAGAG

Table S11. Amino acid sequence of the Ymu1 TnpB bacterial expression construct (related to Methods)

ID	Protein sequence
WT Ymu1	MKSSHHHHHHHHHHGSSMKIEEGKLVIWINGDKGYNGLAEVGKKFEKDTGIKVTVEHPDKLEEKFPQVAATGDGPDIIFWAHDRFGGYAQSGLLAEITPDKAFQDKLYPFTWDAVRYNGKLIAYPIAVEALSLIYNKDLLPNPPKTWEEIPALDKELKAKGKSALMFNLQEPYFTWPLIAADGGYAFKYENGKYDIKDVGVDNAGAKAGLTFLVDLIKNKHMNADTDYSIAEAAFNKGETAMTINGPWAWSNIDTSKVNYGVTVLPTFKGQPSKPFVGVLSAGINAASPNKELAKEFLENYLLTDEGLEAVNKDKPLGAVALKSYEEELAKDPRIAATMENAQKGEIMPNIPQMSAFWYAVRTAVINAASGRQTVDEALKDAQTNSSSNNNNNNNNNNLGIEENLYFQSNAMLQHKAYEYRIYPDKKQETLIAKTIGSSRYVYNHFLELWNKEYEETGKGLTYYACSKLLTKLKRDPETVWLCEVDKFSLQNSLRNLSDAFSRFFKGQNEHPQFKSKKSPRQSYTTQYTNNNIAVSGNCLKLPKLGLVKFADSREMKGRILNATVRRKSSGKFFVSILCEEEICELPKTDSSVGIDLGIIDFAVMSDGSRHDNNHFTRQMEERLRREQRKLARRALAAEKRGISLSEARNYQKQRRKVARLYEKVANQRKEYLNKLSTEIVKNHDIICIEDLNVKGMMRNHKLAKSISDVSWTSLVSKLQYKASWYGKEVIRISRWFPSSQICSECGHKDRKKPLHVREWTCPVCHAHHDRDVNAARNILAEGLRIRALTPGS

10 His-tags; MBP; TEV site; TnpB protein

Table S12. DNA sequence of the 127-nt reRNA expression cassette (related to Methods)

ID	DNA sequence
127-nt reRNA	TAATACGACTCACTATAGGCAAACAGGAACCGCAGGAATTGCGGGGGTAGCTTGGTAAACAAGAGAAACCTCTGCCGGCAAAGAAATAAGCCGGTAAGTATGCTCTGTTCCCAAGAATCTCGTGACTTTAGTCATGAGAGTTTCAATCTTCTGGATTGTTGTGGCCGGCATGGTCCCAGCCTCCTCGCTGGCGCCGGCTGGGCAACATGCTTCGGCATGGCGAATGGGAC

T7 promoter; reRNA scaffold; guide sequence (for Target 1); HDV ribozyme

Table S13. Sequences of all the DNA substrates used in DNA cleavage assays (related to Methods)

Internal ID	Description	Sequence (5′-3′)	Figures
dZZ01	Ymu1 DNA substrate 0-16 bp corresponding to target 1; NTS; Cy5	/5Cy5/CCGGAGGTCATA ATGATTGATTCTTCTGG ATTGTTGTAAGCAGCAT TTGAGCAAAAATCT	Fig. 3C-F, 5H, S1E-F, S3E-J, S5H
dZZ01C	Ymu1 DNA substrate 2-16 bp corresponding to target 1 (substrate pre-unwound at bases 1 and 2) ; NTS; Cy5	/5Cy5/CCGGAGGTCATA ATGATTGATAGTTCTGG ATTGTTGTAAGCAGCAT TTGAGCAAAAATCT	Fig. 5H, S5H
dZZ01F	Ymu1 DNA substrate 0-7 and 10-16 bp corresponding to target 1 (substrate pre-unwound at bases 8 and 9); NTS; Cy5	/5Cy5/CCGGAGGTCATA ATGATTGATAGAAGACC TTTGTTGTAAGCAGCAT TTGAGCAAAAATCT	Fig. 5H, S5H
dZZ01G	Ymu1 DNA substrate 10-16 bp corresponding to target 1 (substrate pre-unwound from bases 1 to 9); NTS; Cy5	/5Cy5/CCGGAGGTCATA ATGATTGATTCTTCTGC CTTGTTGTAAGCAGCAT TTGAGCAAAAATCT	Fig. 5H, S5H
dZZ02	Ymu1 DNA substrate 0-16bp corresponding to target 1; TS; FAM	/56-FAMN/AGATTTTTGCTCA AATGCTGCTTACAACAA TCCAGAAGAATCAATCA TTATGACCTCCGG	Fig. 3C-F, 5H, S1E-F, S3E-J, S5H
dZZ013	Ymu1 DNA substrate 0-16 bp corresponding to target 2; NTS; Cy5	/5Cy5/CCGGAGGTCATA ATGATTGATAAGGCAAA TTCGCCGCAAGCAGCA TTTGAGCAAAAATCT	Fig. 3G-H, S3K-L
dZZ014	Ymu1 DNA substrate 0-16 bp corresponding to target 2; TS; FAM	/56-FAMN/AGATTTTTGCTCA AATGCTGCTTGCGGCG AATTTGCCTTATCAATC ATTATGACCTCCGG	Fig. 3G-H, S3K-L

Table S14. Primer sequences used to generate DNA tether fragments in AuRBT (related to Fig. S4 and Methods).

	Primer Sequence (5′-3′)	Length	Template	Digest
F500	gatcgaagacacttagACAACCCACAAGTATAGAGGCTCCTATG<br><br>GACGCGGATATAATGACATTTCCTAAC	520	pFO-SE2	BbsI
XD	gaagggtctcatgacTCACTATAGGGCGAATTGGAGCTCCACCGCG<br><br>gaagggtctcactaaCACTAAAGGGAACAAAAGCTGGTAC	4165	pFO-SE2	BsaI
SOI	gatcgaagacacctgcTGACGCATCAGTCAGTACTACTGACG<br><br>gatcgaagacacgtcaGTGGTAGCTGCTGTGCGAGTG	290	TnpB Gblock	BbsI

SOI 1-4MM	gatcgaagacacctgcTGACGCATCAGTCAGTACTACTGACG<br><br>gatcgaagacacgtcaGTGGTAGCTGCTGTGCGAGTG	290	TnpB 1-4MMGblock	BbsI
SPMDIG	GCGCAGCACGCAGATACACTC<br><br>gatcgaagacacgcagCATACGTCTGCGTCGCTG	320	C12Gblock	BbsI

Biotin-modified nucleotides highlighted in blue.

Table S15. Target sequences in the SOI (related to Fig. S4 and Methods).

DNA Target	Sequence
Target 1	TTGATTCTTCTGGATTGTTGT
1-4 mismatch	TTGATAGAACTGGATTGTTGT

TAM sequence highlighted in yellow.

Table S16. The full DNA tether sequence with Target 1 (related to Fig. S4 and Methods)

GACGCGGATATAATGACATTTCCTAACTTTTGGGCAAAAATTCGCTATCATATGCGAGAACCGTTT
GCGGAGTTTCTCGGGACACTAGTTCTTGTCATTTTTGGTGTTGGTGGTAATCTTCAAGCAACTGTA
ACAAAAGGTAGTGGTGGTTCCTATGAATCCCTATCATTTGCATGGGGGTTCGGTTGTATGCTTGGT
GTTTACGTCGCAGGCGGTATTAGTGGTGGTCATATTAACCCTGCTGTTACGATTTCAATGGCAATT
TTTCGAAAATTCCCCTGGAAAAAGGTGCCCGTATATATTGTTGCTCAGATTATCGGTGCATATTTTG
GAGGAGCTATGGCTTATGGTTATTTTTGGAGCTCTATCACAGAATTTGAGGGAGGTCCGCACATAA
GAACAACGGCGACCGGTGCGTGTTTGTTTACTGATCCAAAGTCTTACGTCACGTGGAGAAATGCC
TTCTTTGACGAATTCATAGGAGCCTCTATACTTGTGGGTTGTCTAACACTAAAGGGAACAAAAGCT
GGTACCGGGCCCCCCCTCGAGCGGTACCCCACTTACCCACCCCGGAAATTTGAGTTATAAACGTT
GTTTGAGCTTTACCTAGTCTTGGTCGATCAAAAGTTCTGGTACCTTTTCACCATGTCTCCCCCCTTA
TTCATATAAAAAGAAGCGTATAATCGCACAGTATAACGCTCCTCTGATATATGATCTAGACCCAAGT
AATGAGTTACGAATCTGGGAGGTCATCCTCCTCTTCCGAGAGTACACGGCCACCAACGCTAAAAG
AAGAACCTAATGGTAAAATAGCTTGGGAAGAAAGTGTCAAAAAATCTAGGGAAAATAACGAAAATG
ACAGCACTCTCTTGAGGCGAAAGCTAGGTGAGACTCGAAAAGCAATTGAAACTGGAGGATCATCG
AGAAATAAACTTTCTGCTTTGACACCCTTGAAAAAAGTGGTTGACGAGAGGAAGGATTCGGTACAA
CCACAGGTCCCTTCCATGGGTTTTACTTATTCTTTGCCTAATTTGAAGACTTTAAACAGTTTTTCAGA
TGCTGAGCAAGCACGTATAATGCAAGATTATCTATCCAGGGGGGTAAATCAAGGCAACAGTAATAA
TTATGTAGACCCACTATATCGGCAATTAAATCCAACTATGGGTAGTAGCAGGAACAGGCCTGTTTG
GAGTTTAAATCAGCCGTTACCGCATGTATTGGATCGAGGCTTGGCAGCAAAGATGATACAAAAGAA
TATGGATGCAAGGTCCCGCGCATCATCGAGACGAGGGTCGACCGATATTTCAAGGGGGGGTTCTA
CTACGTCAGTGAAAGACTGGAAAAGGCTCCTTAGAGGTGCAGCACCGGGTAAAAAGCTTGGTGAC
ATCGAAGCTCAAACGCAACGCGATAATACTGTTGGTGCAGATGTGAAACCTACTAAGTTAGAGCCT
GAAAACCCACAAAAGCCCTCTAACACGCATATTGAGAATGTTTCACGTAAGAAAAAGCGTACTTCG
CATAATGTCAATTTTTCATTAGGCGATGAAAGCTACGCATCCTCCATAGCCGATGCAGAATCCAGA
AAATTAAAGAACATGCAAACCCTCGATGGTTCTACTCCGGTTTATACGAAGCTTCCTGAAGAACTTA
TTGAAGAGGAAAATAAAAGTACGAGTGCATTAGATGGTAATGAAATTGGTGCCTCAGAAGATGAAG
ACGCGGATATAATGACATTTCCTAACTTTTGGGCAAAAATTCGCTATCATATGCGAGAACCGTTTGC
GGAGTTTCTCGGGACACTAGTTCTTGTCATTTTTGGTGTTGGTGGTAATCTTCAAGCAACTGTAACA
AAAGGTAGTGGTGGTTCCTATGAATCCCTATCATTTGCATGGGGGTTCGGTTGTATGCTTGGTGTT
TACGTCGCAGGCGGTATTAGTGGTGGTCATATTAACCCTGCTGTTACGATTTCAATGGCAATTTTT
CGAAAATTCCCCTGGAAAAAGGTGCCCGTATATATTGTTGCTCAGATTATCGGTGCATATTTTGGA
GGAGCTATGGCTTATGGTTATTTTTGGAGCTCTATCACAGAATTTGAGGGAGGTCCGCACATAAGA
ACAACGGCGACCGGTGCGTGTTTGTTTACTGATCCAAAGTCTTACGTCACGTGGAGAAATGCCTTC
TTTGACGAATTCATAGGAGCCTCTATACTTGTGGGTTGTTTGATGGCGCTATTGGATGATAGTAAT
GCTCCACCTGGCAATGGTATGACCGCATTAATTATTGGATTCTTAGTCGCTGCAATTGGTATGGCC
CTTGGATATCAAACAAGTTTCACAATCAATCCTGCAAGAGATCTCGGTCCTCGCATATTTGCTTCCA
TGATTGGCTATGGTCCACATGCTTTTCATCTCACACATTGGTGGTGGACATGGGGAGCCTGGGGT

GGTCCAATTGCCGGCGGTATTGCTGGAGCACTCATATATGACATTTTCATTTTTACTGGATGCGAA
TCCCCAGTCAACTACCCAGACAACGGTTATATTGAGAATAGGGTAGGCTGCAGCCCGGGGGATCC
ACTAGTTCTAGAGCGGCCGCCGGTACCCCACTTACCCACCCCGGAAATTTGAGTTATAAACGTTGT
TTGAGCTTTACCTAGTCTTGGTCGATCAAAAGTTCTGGTACCTTTTCACCATGTCTCCCCCCTTATT
CATATAAAAAGAAGCGTATAATCGCACAGTATAACGCTCCTCTGATATATGATCTAGACCCAAGTAA
TGAGTTACGAATCTGGGAGGTCATCCTCCTCTTCCGAGAGTACACGGCCACCAACGCTAAAAGAA
GAACCTAATGGTAAAATAGCTTGGGAAGAAAGTGTCAAAAAATCTAGGGAAAATAACGAAAATGAC
AGCACTCTCTTGAGGCGAAAGCTAGGTGAGACTCGAAAAGCAATTGAAACTGGAGGATCATCGAG
AAATAAACTTTCTGCTTTGACACCCTTGAAAAAAGTGGTTGACGAGAGGAAGGATTCGGTACAACC
ACAGGTCCCTTCCATGGGTTTTACTTATTCTTTGCCTAATTTGAAGACTTTAAACAGTTTTTCAGATG
CTGAGCAAGCACGTATAATGCAAGATTATCTATCCAGGGGGGTAAATCAAGGCAACAGTAATAATT
ATGTAGACCCACTATATCGGCAATTAAATCCAACTATGGGTAGTAGCAGGAACAGGCCTGTTTGGA
GTTTAAATCAGCCGTTACCGCATGTATTGGATCGAGGCTTGGCAGCAAAGATGATACAAAAGAATA
TGGATGCAAGGTCCCGCGCATCATCGAGACGAGGGTCGACCGATATTTCAAGGGGGGGTTCTAC
TACGTCAGTGAAAGACTGGAAAAGGCTCCTTAGAGGTGCAGCACCGGGTAAAAAGCTTGGTGACA
TCGAAGCTCAAACGCAACGCGATAATACTGTTGGTGCAGATGTGAAACCTACTAAGTTAGAGCCTG
AAAACCCACAAAAGCCCTCTAACACGCATATTGAGAATGTTTCACGTAAGAAAAAGCGTACTTCGC
ATAATGTCAATTTTTCATTAGGCGATGAAAGCTACGCATCCTCCATAGCCGATGCAGAATCCAGAA
AATTAAAGAACATGCAAACCCTCGATGGTTCTACTCCGGTTTATACGAAGCTTCCTGAAGAACTTAT
TGAAGAGGAAAATAAAAGTACGAGTGCATTAGATGGTAATGAAATTGGTGCCTCAGAAGATGAAGA
CGCGGATATAATGACATTTCCTAACTTTTGGGCAAAAATTCGCTATCATATGCGAGAACCGTTTGC
GGAGTTTCTCGGGACACTAGTTCTTGTCATTTTTGGTGTTGGTGGTAATCTTCAAGCAACTGTAACA
AAAGGTAGTGGTGGTTCCTATGAATCCCTATCATTTGCATGGGGGTTCGGTTGTATGCTTGGTGTT
TACGTCGCAGGCGGTATTAGTGGTGGTCATATTAACCCTGCTGTTACGATTTCAATGGCAATTTTT
CGAAAATTCCCCTGGAAAAAGGTGCCCGTATATATTGTTGCTCAGATTATCGGTGCATATTTTGGA
GGAGCTATGGCTTATGGTTATTTTTGGAGCTCTATCACAGAATTTGAGGGAGGTCCGCACATAAGA
ACAACGGCGACCGGTGCGTGTTTGTTTACTGATCCAAAGTCTTACGTCACGTGGAGAAATGCCTTC
TTTGACGAATTCATAGGAGCCTCTATACTTGTGGGTTGTTTGATGGCGCTATTGGATGATAGTAAT
GCTCCACCTGGCAATGGTATGACCGCATTAATTATTGGATTCTTAGTCGCTGCAATTGGTATGGCC
CTTGGATATCAAACAAGTTTCACAATCAATCCTGCAAGAGATCTCGGTCCTCGCATATTTGCTTCCA
TGATTGGCTATGGTCCACATGCTTTTCATCTCACACATTGGTGGTGGACATGGGGAGCCTGGGGT
GGTCCAATTGCCGGCGGTATTGCTGGAGCACTCATATATGACATTTTCATTTTTACTGGATGCGAA
TCCCCAGTCAACTACCCAGACAACGGTTATATTGAGAATAGGGTAGGGGCCGCCACCGCGGTGG
AGCTCCAATTCGCCCTATAGTGAGTCAGTGGTAGCTGCTGTGCGAGTGTATCTGCGTGCTGCGCT
AGTGAGATCACGTGCTCATGATACGTGATCTCGTCGCGTAGTGCAGCGAGATCGCGTATGTACTG
CTT**ACAACAATCCAGAAGA**ATCAAGCAGCGATATCTCGTGTGCAGACAGCTCGCGACGATGCGC
TACGTCATCTAGTAGTGCGTACTGACTCTCAGTATACGACATATCTAGACGCTGCTGACTGTATAT
CTCTATAGCTCAGGCATGCTAGTCTAGCGTCAGTAGTACTGACTGATGCGTCAGCAGCATACGTCT
GCGTCGCTGTGGTGTATACGTGCGACATACTCAGAGTCGCGCTCTCTACGGTGTCACTCGAGCTG
TAGATGCTCAGTGCGATACAGTACGCTCCATGCTGCTCATCAGCTGCTCAGCTCTGTATCTGCTCT
GACATGACGTGACTCAGCGCGAGCGTATGATCGAGATACTGATCGTACACTCACTGGTCAGTGTG
TAGCGTGACTGCGTGCAGCTCAGTCAGAGCATCACTCCTCGCCATCATGTCCGAGACTAGTGGTG
GTAGCTGCTGTGCGAGTGTATCTGCGTGCTGCGC

A represents adenine nucleotides paired with biotin-modified thymines, where the streptavidin-coated gold rotor bead can be attached;
bolded region represents the 16-nt Target 1 sequence (the TS sequence);
yellow highlighted region represents the TAM (the complementary sequence of 5′-TTGAT);
cyan highlighted region represents the segment with multiple incorporated dUTP-digoxigenin (Roche);
green highlighted region represents the segment with multiple incorporated fluorescein-dUTPs (Roche).

Table S17. AF3 query sequences for the WT Ymu1 ternary complex (related to Methods)

	Sequences

Protein	MLQHKAYEYRIYPDKKQETLIAKTIGSSRYVYNHFLELWNKEYEETGKGLTYYACSKL<br>LTKLKRDPETVWLCEVDKFSLQNSLRNLSDAFSRFFKGQNEHPQFKSKKSPRQSYT<br>TQYTNNNIAVSGNCLKLPKLGLVKFADSREMKGRILNATVRRKSSGKFFVSILCEEEI<br>CELPKTDSSVGIDLGIIDFAVMSDGSRHDNNHFTRQMEERLRREQRKLARRALAAEK<br>RGISLSEARNYQKQRRKVARLYEKVANQRKEYLNKLSTEIVKNHDIICIEDLNVKGMM<br>RNHKLAKSISDVSWTSLVSKLQYKASWYGKEVIRISRWFPSSQICSECGHKDRKKPL<br>HVREWTCPVCHAHHDRDVNAARNILAEGLRIRALTPGS
RNA	CCGCACCCCCUGCGGGGGUAGCUUGGUAAACAAGAGAAACCUCUGCCGGCAA<br>AGAAAUAAGCCGGUAAGUAUGCUCUGUUCCCAAGAAUCUCGUGACUUUAGUCA<br>UGAGAGGGGGGAUCUUCUGGAUUGUU
DNA	AACAATCCAGAAGAATCAATCA
DNA	TGATTGAT

References

1. Wiggins, P.A. An information-based approach to change-point analysis with applications to biophysics and cell biology. *Biophys J* **109**, 346-54 (2015).
2. LaMont, C.H. & Wiggins, P.A. The Development of an Information Criterion for Change-Point Analysis. *Neural Computation* **28**, 594-612 (2016).
3. Sasnauskas, G. et al. TnpB structure reveals minimal functional core of Cas12 nuclease family. *Nature* **616**, 384-389 (2023).
4. Nakagawa, R. et al. Cryo-EM structure of the transposon-associated TnpB enzyme. *Nature* **616**, 390-397 (2023).
5. Skopintsev, P. et al. Structure and evolution-guided design of minimal RNA-guided nucleases. *bioRxiv*, 2025.12.08.692503 (2025).
6. Ovchinnikov, S., Kamisetty, H. & Baker, D. Robust and accurate prediction of residue-residue interactions across protein interfaces using evolutionary information. *Elife* **3**, e02030 (2014).
7. Stella, S. et al. Conformational Activation Promotes CRISPR-Cas12a Catalysis and Resetting of the Endonuclease Activity. *Cell* **175**, 1856-1871 e21 (2018).
8. Pausch, P. et al. DNA interference states of the hypercompact CRISPR–CasΦ effector. *Nature Structural & Molecular Biology* **28**, 652-661 (2021).
9. Guan, K. et al. Comparative characterization of Cas12f orthologs reveals mechanistic features underlying enhanced genome editing efficiency. *bioRxiv*, 2025.08.14.670346 (2025).
10. Hino, T. et al. An AsCas12f-based compact genome-editing tool derived by deep mutational scanning and structural analysis. *Cell* **186**, 4920-4935 e23 (2023).
11. Thornton, B.W. et al. Mutational scanning of TnpB reveals latent activity for genome editing. *bioRxiv* (2025).
12. Weiss, T. et al. Viral delivery of an RNA-guided genome editor for transgene-free germline editing in Arabidopsis. *Nature Plants* (2025).
